# Regulation of SLC7A11 by LncRNA GPRC5D-AS1 mediates ferroptosis in skeletal muscle: Mechanistic exploration of sarcopenia

**DOI:** 10.3389/fmolb.2025.1557218

**Published:** 2025-04-16

**Authors:** Wei Gong, Yan Wang, Qun Li, Yating Gao, Jie Li

**Affiliations:** ^1^ Department of Critical Care Medicine, The First Hospital of Jilin University, Changchun, China; ^2^ Department of Geriatrics and Special medical treatment, The First Hospital of Jilin University, Changchun, China; ^3^ Health Examination Center, The First Hospital of Jilin University, Changchun, China

**Keywords:** ferroptosis, long non-coding RNA, GPRC5D-AS1, SLC7A11, sarcopenia

## Abstract

Sarcopenia is a chronic, progressive disease characterized by the gradual loss of skeletal muscle strength and mass. This study investigates the role of the long non-coding RNA GPRC5D-AS1 in the development and progression of sarcopenia through its regulation of *SLC7A11*. Skeletal muscle samples were obtained from sarcopenia patients and healthy controls to assess the expression levels of GPRC5D-AS1 and *SLC7A11*. Flow cytometry was used to evaluate iron content, lipid peroxidation, and antioxidant markers. A ferroptosis model was established in human skeletal muscle cells (HSKM) using the inducer erastin, and GPRC5D-AS1 overexpression plasmids were introduced to observe their effects on cell proliferation and ferroptosis indicators. In the sarcopenia group, both GPRC5D-AS1 and *SLC7A11* expression levels decreased significantly, along with SLC7A11 protein translation. Erastin treatment markedly reduced cell viability and increased iron content, elevating ferroptosis marker genes (*COX2, ACSL4, PTGS2, NOX1*) while reducing *GPX4* and *FTH1* levels. The overexpression of GPRC5D-AS1 reversed these changes, enhancing antioxidant capacity and cell survival. Conversely, silencing *SLC7A11* diminished the protective effects of GPRC5D-AS1 on cell proliferation and ferroptosis. These findings suggest that GPRC5D-AS1 overexpression increases *SLC7A11* expression and reduces ferroptosis incidence in HSKM.

## Introduction

Sarcopenia is a chronic and progressive condition characterized by the loss of skeletal muscle strength and mass, distinct from the physiological process of muscle atrophy (Cruz-Jentoft et al., 2019; Feike et al., 2021). It is age-related and predominantly observed in older adults, often resulting in unexpected falls, fractures, and disabilities that diminish mobility and the ability to carry out daily activities. This condition imposes a significant burden on individuals, families, and society. The pathophysiological basis of sarcopenia is primarily linked to a reduction in type II muscle fibres (Damluji et al., 2023); however, a consensus on specific underlying mechanisms remains elusive. As such, research into the mechanisms of sarcopenia is essential for guiding its prevention and treatment.

Ferroptosis is a distinctive form of iron-dependent, non-apoptotic regulated cell death, characterized by mitochondrial shrinkage and increased mitochondrial membrane density (Yang and Stockwell, 2016). This process arises from the disruption of reduced glutathione (GSH)-dependent antioxidant defense mechanisms and the subsequent accumulation of lipid peroxides (Dixon et al., 2012; Tang et al., 2018). Intracellular iron overload leads to a rapid increase in reactive oxygen species (ROS), which is a significant trigger for ferroptosis (Chinnery et al., 2003). The build-up of lipid peroxidation products and the depletion of polyunsaturated fatty acids in cell membranes are crucial contributors to cell death (Yang and Stockwell, 2016). Research has highlighted the important role of ferroptosis in sarcopenia. A study involving 23 older participants and 11 healthy young controls assessed iron content, transferrin levels, and oxidative stress markers in skeletal muscle tissues. The results indicated that iron levels in the skeletal muscle of the older group were significantly higher than in the controls, alongside decreased transferrin levels and a marked increase in oxidative stress markers, suggesting a close relationship between iron metabolism and skeletal muscle ageing (Picca et al., 2019).

Solute carrier family 7 member 11 (SLC7A11), as the light chain component of the cellular membrane system Xc^−^, plays a crucial role in the transport of glutamate and cysteine, thereby influencing GSH production. GSH is a vital antioxidant that helps cells combat oxidative stress and lipid peroxidation, which is essential for maintaining cellular homeostasis and preventing ferroptosis (Cancer Genome Atlas Research Network, 2012; Koppula et al., 2021). Abnormal functioning of *SLC7A11* has been linked to the onset and progression of various diseases, including cancer, impaired wound healing in diabetes, and neurodegenerative disorders (Mesci et al., 2015; Zhang et al., 2018; Maschalidi et al., 2022). In skeletal muscle tissues of aging-prone mice (SAMP8), *SLC7A11* expression has been significantly reduced. Furthermore, the introduction of p53 has been shown to markedly enhance *SLC7A11* expression, improving the survival rate of skeletal muscle cells *in vitro* and inhibiting ferroptosis (Huang et al., 2021).

Long non-coding RNAs (lncRNAs) are RNA molecules longer than 200 nucleotides that do not encode proteins. They play a regulatory role in various cellular processes, including gene transcription, mRNA translation, and protein transport, and are implicated in the pathological mechanisms of a range of diseases such as cancer, cardiovascular conditions, and viral infections (Liu et al., 2023; Banerjee et al., 2024). Research suggests that lncRNAs can influence ferroptosis via the regulation of *SLC7A11* expression, thereby affecting disease progression. For example, ADAMTS9-AS1 is highly expressed in ovarian cancer, where it produces antioxidant substances that reduce ferroptosis in cancer cells, promoting tumor growth and proliferation (Cai et al., 2022). Similarly, SLC16A1-AS1 exhibits increased expression levels in renal cancer tissues, with its expression correlating with overall survival rates in patients. Silencing SLC16A1-AS1 significantly impairs the viability, proliferation, and migration of renal cancer cells (Li et al., 2022). Additionally, elevated levels of NRAV expression are associated with poor prognosis in liver cancer patients, and *in vitro* studies indicate that knockdown of NRAV markedly inhibits the proliferation and migration of liver cancer cells (Zong et al., 2024).

Research into the influence of lncRNAs on the ferroptosis mechanism in skeletal muscle cells, and their subsequent effect on the development of sarcopenia, remains limited. Our previous studies have demonstrated that the transcription and translation levels of GPRC5D-AS1 are significantly reduced in the skeletal muscle tissues of the older adults (mean age 79.33 ± 0.58), compared to those from healthy young individuals (Zheng et al., 2021). This observation prompted us to hypothesis that GPRC5D-AS1 may play a crucial role in mitigating skeletal muscle ageing. Accordingly, this study aims to investigate whether GPRC5D-AS1 influences the occurrence of ferroptosis through the regulation of *SLC7A11* expression, thereby significantly affecting the onset and progression of sarcopenia.

## Materials and methods

### Collection of skeletal muscle tissue from clinical patients

This study received ethical approval from the Ethics Committee of the First Hospital of Jilin University (ethical number: 19K083-001), and all participants provided informed consent. Clinical data are detailed in the Supplementary Table S1. We adhered to the diagnostic criteria for sarcopenia as defined in the 2019 *Sarcopenia: revised European consensus on definition and diagnosis.* From the Department of Geriatrics and Special Medical Treatment, we selected five patients who met the sarcopenia criteria for skeletal muscle tissue sampling, alongside samples from five healthy young individuals for comparison. All samples were immediately placed in RNA stabilizer tubes to maintain RNA integrity and stored at 4°C before transferring to −80°C for long-term storage. The samples were thawed on ice, washed with saline, and then cut into small pieces and frozen in liquid nitrogen for RNA and protein extraction. Subsequent analyses will assess target RNA expression levels and conduct Western blot analysis for protein expression of target genes.

### Cell Culture

Human Skeletal Muscle Myoblasts (HSKM) were obtained from Cellverse (Shanghai, China) and cultured according to the manufacturer’s protocol. Upon receipt, the primary cells were stored at −80°C until thawed in a 37°C water bath. After centrifugation, the cells were resuspended in pre-warmed specific culture medium (iCell-0086a-001b, Cellverse) and transferred to culture flasks for incubation at 37°C in a 5% CO_2_ atmosphere. The culture medium was changed every 48 h while monitoring cell morphology and growth density. Passaging occurred when cell density exceeded 90%. The thawed primary cells were stabilized and passaged up to the third generation before conducting further experiments and cryopreserving the seed cells.

### Cell transfection

Cultured cells were resuspended in complete medium without penicillin-streptomycin, and cell counts were performed. A total of 4 × 10^4^ cells were seeded in each well of a 24-well plate and incubated overnight. Following the manufacturer’s instructions, serum-free Opti-MEM medium containing Lipofectamine 2000 (solution A) and serum-free Opti-MEM with the target plasmid or siRNA (solution B) were prepared. Both solutions were allowed to stand for 5 min before being combined and incubated at room temperature for 20 min to form the transfection mixture. This mixture was added to the 24-well plate, and after 6 h, the medium was replaced with fresh complete medium for an additional 48 h. Transfection efficiency was assessed using qRT-PCR.

### Cell counting kit-8 (CCK-8) assay

Collected cells were counted, resuspended to a density of 1 × 10^4^ cells per well, and plated in a 96-well plate with six replicates for each group. Phosphate-buffered saline (PBS) was added around the wells to prevent evaporation. The plate was incubated overnight at 37°C in a 5% CO_2_ atmosphere before transfection the following day. After transfection, the medium was replaced, and the cells were incubated for an additional 24, 48, or 72 h. At the end of the incubation, 10 μl of CCK-8 solution (C0038, Beyotime) was added to each well, and the plate was incubated for 1–4 h (OD ≤ 2.0). Optical density was measured with a microplate reader, and cell viability curves were plotted.

### Flow cytometry analysis

#### Analysis of intracellular iron content

Processed cells were collected, washed with PBS, and digested with trypsin before being transferred to centrifuge tubes. After centrifugation, the cells were washed with pre-warmed PBS, and the supernatant was discarded. Each sample was resuspended in PBS, using a larger volume for the blank control. FeRhoNox-1 dye (GC901, Goryo Chemical) was added to the experimental groups at a final concentration of 5 μM, thoroughly mixed, and incubated in the dark at 37°C in a CO_2_ incubator for 60 min. After incubation, cells were washed with pre-warmed PBS, centrifuged, and resuspended in PBS. Samples were analyzed with a flow cytometer using an excitation wavelength of 532 nm and a detection wavelength of 570 nm to evaluate fluorescence intensity and intracellular iron content.

#### Analysis of cellular lipid peroxidation levels

Processed cells were collected, washed with PBS, and digested with trypsin to create a suspension, which was transferred to centrifuge tubes. After centrifugation, the cells were washed with pre-warmed PBS, and the supernatant was discarded. Each sample was resuspended in PBS, with a larger volume used for the blank control. BODIPY (581/591) dye (D3861, Invitrogen) was added to the experimental groups, mixed, and incubated in the dark at 37°C in a CO_2_ incubator. Following incubation, cells were washed, centrifuged, and resuspended in PBS. The samples were analyzed with a flow cytometer at an excitation wavelength of 488 nm and an emission wavelength of 525 nm to evaluate fluorescence intensity and lipid peroxidation levels.

#### Quantitative real-time PCR (qRT-PCR) analysis

Total RNA was extracted from cell or tissue samples using TRIzol reagent (9109, Takara) as per the manufacturer’s instructions. RNA concentration and purity were assessed with a spectrophotometer to ensure the 260/280 ratio was within the acceptable range. A reverse transcription reaction was then performed to convert RNA to cDNA using PrimeScript RT Master MIX (RR036A, TaKaRa), following the manufacturer’s guidelines. For qRT-PCR, Power SYBR Green PCR Master Mix (4367659, Thermo) was used, including cDNA, SYBR Green Master Mix, and specific primers. The PCR programme comprised an initial denaturation step followed by amplification cycles. All samples were run in triplicate, and relative gene expression levels were calculated using the ΔΔCt method with *GAPDH* as the internal control. Reactions were conducted on a quantitative fluorescent PCR instrument. Primer sequences were as shown in Table 1.

**TABLE 1:** The primer sequences for mRNAs and long non-coding RNA (lncRNA).

Primers	Sequence (5′-3′)
GPRC5D-AS1-F	GCGTTCCTTAGAGAAATGGCTA
GPRC5D-AS1-R	ACACAGCTCCAGTAGTCGTTGA
SLC7A11-F	TCTCCAAAGGAGGTTACCTGC
SLC7A11-R	AGACTCCCCTCAGTAAAGTGAC
NOX1-F	TTGTTTGGTTAGGGCTGAATGT
NOX1-R	GCCAATGTTGACCCAAGGATTTT
COX2-F	GAGATGATCTACCCTCCTCAAGTC
COX2-R	AGTATTAGCCTGCTTGTCTGGAAC
ACSL4-F	CATCCCTGGAGCAGATACTCT
ACSL4-R	TCACTTAGGATTTCCCTGGTCC
GPX4-F	GAGGCAAGACCGAAGTAAACTAC
GPX4-R	CCGAACTGGTTACACGGGAA
FTH1-F	CCCCCATTTGTGTGACTTCAT
FTH1-R	GCCCGAGGCTTAGCTTTCATT
PTGS2-F	ATGCTGACTATGGCTACAAAAGC
PTGS2-R	TCGGGCAATCATCAGGCAC

#### Western blot (WB)

Samples were lysed with RIPA buffer containing protease and phosphatase inhibitors. After ultrasonic treatment and centrifugation, the supernatant was collected, and protein concentration determined. SDS-PAGE was conducted using 20 µg of protein per sample, followed by transfer to a PVDF membrane (IPVH00010, Millipore). The membrane was blocked with non-fat dry milk to minimize non-specific binding and then incubated overnight at 4°C with a primary antibody targeting the protein of interest and an internal control antibody. After washing, a labelled secondary antibody was added and incubated at room temperature. Chemiluminescent detection was used to visualize protein signals, and optical density was analyzed with ImageJ software. *GAPDH* signal intensity was used as the normalization reference for target protein expression levels were normalized. All experiments were performed in triplicate, and results are presented as mean ± standard deviation, with statistical analysis conducted using statistical software.

#### Other biochemical assays and experiments

##### Superoxide dismutase (SOD) assay

The SOD detection kit (A001-3, Nanjing Jiancheng) was utilized. Cells from each group were digested with trypsin and collected, followed by the addition of pre-chilled PBS for homogenization. Protein concentration was quantified using the BCA method. Samples and standards were added to a microplate, followed by BCA working solution and incubation to determine absorbance. A standard curve was constructed to calculate protein concentrations. Samples were mixed and incubated at 37°C for 20 min, and absorbance was measured at 450 nm using a microplate reader to calculate SOD activity.

##### Malondialdehyde (MDA) assay

The MDA detection kit (S0131S, Beyotime) was used. Lysis buffer served as a blank control, alongside various concentrations of standard solutions and the test samples. MDA detection working solution was added, mixed thoroughly, and heated at 100°C for 15 min. After cooling to room temperature, the mixture was centrifuged, and the supernatant was transferred to a 96-well plate. Absorbance was measured at 532 nm using a microplate reader to evaluate MDA content in the samples.

##### RNA pull-down analysis

Samples were divided into three groups. The Input group contained total proteins extracted from the cell lysate, serving as a reference. The Sense group included a sense RNA probe for GPRC5D-AS1, which was incubated with the proteins to capture specifically bound proteins. The Antisense group utilized an antisense RNA probe for GPRC5D-AS1 as a control for non-specific binding. The sense and anti-sense probes were prepared by *in vitro* transcription, with the sense probe derived from the positive strand of the lncRNA GPRC5D-AS1 transcript and the anti-sense probe derived from the negative strand, both incorporating T7 promoter sequences at their 5' ends to initiate transcription. Unbound proteins were removed through a series of washing steps. Finally, WB analysis was performed on the bound proteins to confirm their interaction with the target RNA. Control groups were included to ensure result reliability.

##### RNA Immunoprecipitation (RIP) analysis

In the RIP experiment, samples were divided into three groups. The Input group involved total RNA and protein extraction from the cell lysate as a reference. The RIP group was incubated with an *SLC7A11* specific antibody (26864-1-AP, Proteintech) and the cell lysate, followed by washing to remove unbound components. The Control group used a non-specific IgG antibody for assessing non-specific binding. RNA enrichment in each group was analyzed by qRT-PCR, and WB was utilized to confirm specific proteins bound to the target RNA.

### Statistical analysis

All experimental data were presented as continuous variables, with results expressed as mean ± standard deviation. Raw data were processed and imported into GraphPad Prism 8 for graphical representation and statistical analysis. WB image data were analyzed for grey values using ImageJ, and the results were input into GraphPad Prism 8 for further processing. Differences between groups were assessed using One-way ANOVA, with a significance level set at *p* < 0.05 for all tests, indicating statistically significant differences.

## Results

### Reduced expression of lncRNA GPRC5D-AS1 and *SLC7A11* in skeletal muscle tissues of sarcopenia patients

**FIGURE 1:**
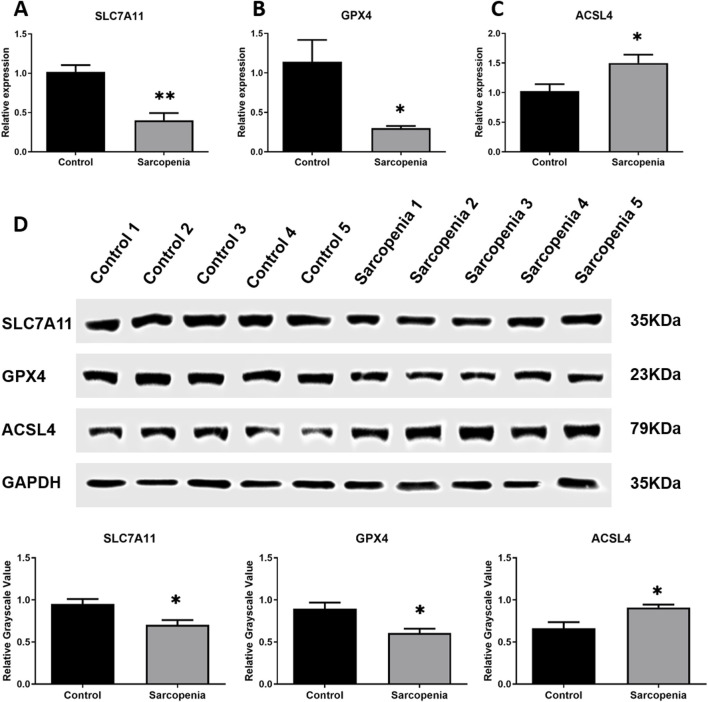
Integrated analysis of *SLC7A11*, *GPX4*, and*ACSL4* in skeletal muscle samples from clinical patients. **(A–C)** qRT-PCR results of *SLC7A11* **(A)**, *GPX4* **(B)**, and*ACSL4* **(C)** mRNA expression levels normalized to *GAPDH*. **(D)** Western blot analysis of corresponding protein expression with *GAPDH* as loading control: representative immunoblots (upper) and relative grayscale values (lower). * or ** indicates a significant difference (*p* < 0.05) or a highly significant difference (*p* < 0.01) compared to the Control group.

The expression levels of GPRC5D-AS1 and *SLC7A11* in the skeletal muscle tissues of sarcopenia patients were evaluated, with GPRC5D-AS1 assessed at the transcriptional level by qRT-PCR and *SLC7A11* analyzed using both qRT-PCR and WB to examine its expression at transcriptional and translational levels, respectively. The qRT-PCR results demonstrated that genes associated with ferroptosis exhibited significantly lower expression of *SLC7A11* and *GPX4* in sarcopenic tissues compared to healthy controls (Figures 1A, B), while *ACSL4* was markedly upregulated. Additionally, Supplementary Figure S1 includes supplementary data on other ferroptosis-related genes, showing that *COX2* expression was also significantly elevated in the sarcopenic group. These mRNA expression patterns align with existing literature. Although *FTH1*, *PTGS2*, and *NOX1* showed no significant differences, their overall trends were consistent with previous reports. WB analysis corroborated qRT-PCR findings, revealing low protein level of SLC7A11 and GPX4 in sarcopenic patients, alongside high expression of ACSL4, with significant differences between groups (Figure 1D). Iron content analysis revealed significantly higher iron levels in the sarcopenic group (Supplementary Figure S1E). Overall, skeletal muscle samples from sarcopenic patients exhibited increased iron levels and notable differences in ferroptosis-related gene markers compared to normal tissues, suggesting ferroptosis may contribute to sarcopenia onset and progression.

### Prediction and validation of the binding interaction between GPRC5D-AS1 and *SLC7A11*

**FIGURE 2:**
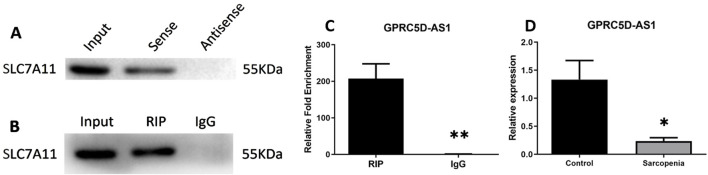
Mechanistic and clinical validation of GPRC5D-AS1/SLC7A11 interactions. **(A)** RNA pull-down analysis demonstrating direct binding between GPRC5D-AS1 sense probe and SLC7A11 protein. **(B, C)** RIP assay of GPRC5D-AS1 in SLC7A11-immunoprecipitated complexes [**(B)** blot image, **(C)** quantification]. **(D)** qRT-PCR analysis of GPRC5D-AS1 expression in skeletal muscle tissues from sarcopenic patients versus healthy controls (normalized to *GAPDH*). * or ** indicates a significant difference (*p* < 0.05) or a highly significant difference (*p* < 0.01) compared to the Control group (or RIP).

Interactions between GPRC5D-AS1 and *SLC7A11* were predicted using public databases and validated through molecular biology experiments. Analysis via LncTar software indicated that GPRC5D-AS1 interacts with SLC7A11 mRNA at positions 1-1743 and 1056-2798, respectively. Additionally, RPISeq analysis revealed the RNA-protein interaction probability of 0.7, indicating a potential interaction between GPRC5D-AS1 and *SLC7A11*. RNA Pull-Down experiments confirmed that the GPRC5D-AS1 sense probe binds to SLC7A11 protein (Figure 2A). Furthermore, RIP showed that after immunoprecipitating *SLC7A11*, GPRC5D-AS1 was enriched by 207.18-fold (p < 0.01) (Figures 2B, C). Critically, qRT-PCR analysis of clinical samples demonstrated that GPRC5D-AS1 expression was significantly reduced in sarcopenic patients’ skeletal muscle tissues compared to healthy controls (Figure 2D), providing pathophysiological relevance to the observed molecular interactions. These findings support the predicted interactions and inform subsequent cellular experiments to construct a skeletal muscle ferroptosis model and further elucidate the regulatory relationship.

### Development of skeletal muscle atrophy and ferroptosis models: Induction of cellular ferroptosis by erastin through lipid peroxidation

**FIGURE 3:**
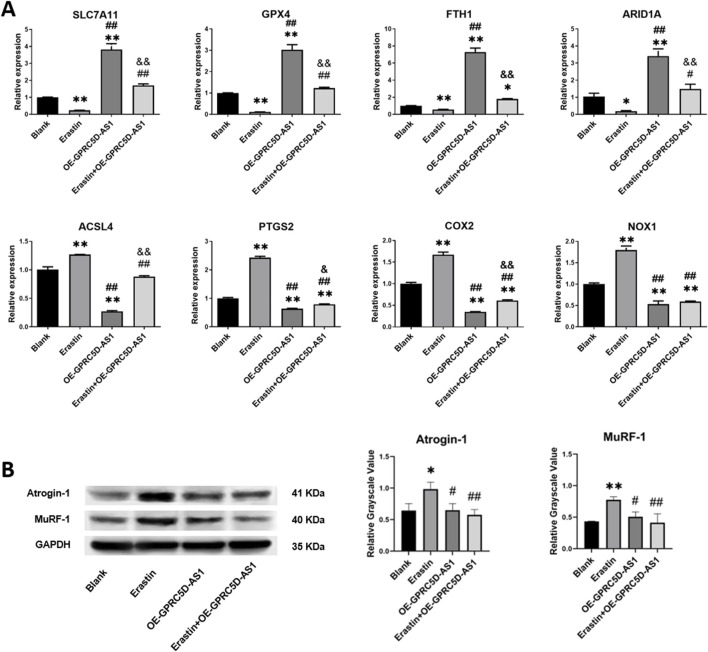
Erastin induces ferroptosis-related gene expression changes and muscle atrophy marker upregulation in HSKM cells. **(A)** qRT-PCR analysis of mRNA levels for ferroptosis-associated genes (normalized to GAPDH). **(B)** Western blot analysis of skeletal muscle atrophy markers, *Atrogin-1* and *MuRF-1*, with *GAPDH* as loading control: representative immunoblots (Left) and quantified relative grayscale values (Right). * or ** indicates a significant difference (*p* < 0.05) or a highly significant difference (*p* < 0.01) compared to the Blank group. # or ## denotes significant differences (*p* < 0.05) or highly significant differences (*p* < 0.01) compared to the Erastin group. & or && indicates significant differences (*p* < 0.05) or highly significant differences (*p* < 0.01) compared to the OE-GPRC5D-AS1 group.

*SLC7A11*, a critical intracellular regulator of lipid peroxidation and suppressor of ferroptosis, serves as a hallmark biomarker for ferroptosis. As shown in Figure 3A, qRT-PCR analysis revealed a significant reduction in *SLC7A11* mRNA levels in the erastin-treated group, aligning with observations in clinical skeletal muscle tissues from patients. Similar downregulation patterns were observed for other ferroptosis-related genes, including *GPX4*, *FTH1*, and *ARID1A*. Conversely, mRNA expression of *ACSL4*, *PTGS2*, *COX2*, and *NOX1* was markedly upregulated. Erastin is a well-established agent for inducing ferroptosis in cells (Zhao et al., 2020; Zhang et al., 2019). Results from the CCK-8 assay demonstrated that erastin inhibits HSKM, with effects becoming more pronounced with increased concentration and exposure time (Supplementary Figures S2A–C). Consequently, a concentration of 0.5 μM erastin was selected for 48 h to induce ferroptosis in subsequent experiments. FeRhoNox-1 levels were significantly elevated in the Erastin group compared to the Blank group (Supplementary Figure S2D), confirming iron accumulation. To evaluate whether erastin-induced ferroptosis mechanistically links to sarcopenia pathophysiology, we analyzed protein expression of *Atrogin-1* and *MuRF-1*, which established biomarkers of skeletal muscle atrophy (Gumucio and Mendias, 2013; Hong et al., 2019). Western blotting demonstrated pronounced upregulation of both proteins in the Erastin group versus controls (Figure 3B), suggesting ferroptosis activation may exacerbate muscle wasting pathways in sarcopenia.

### GPRC5D-AS1 overexpression improves cell viability and decreases membrane lipid peroxidation in ferroptosis cell models

**FIGURE 4:**
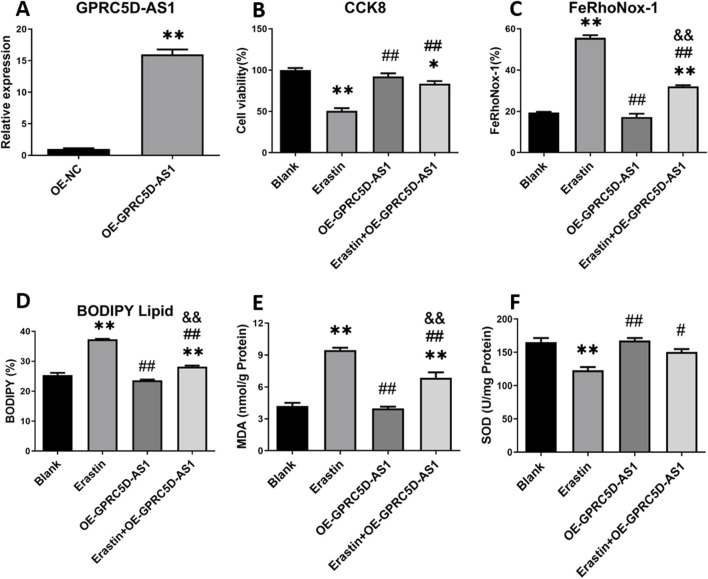
Cell viability and ferroptosis-related marker detection following GPRC5D-AS1 overexpression in HSKM cells. **(A)** Validation of GPRC5D-AS1 overexpression via qRT-PCR. **(B)** Cell viability post-transfection with the GPRC5D-AS1 plasmid. **(C)** Intracellular iron levels after transfection. **(D)** Membrane lipid peroxidation levels following GPRC5D-AS1 overexpression. **(E)** Changes in malondialdehyde (MDA) levels post-transfection. **(F)** Variation in superoxide dismutase (SOD) levels after GPRC5D-AS1 overexpression. * or ** indicates a significant (*p* < 0.05) or highly significant difference (*p* < 0.01) compared to the Blank group. # or ## denotes significant (*p* < 0.05) or highly significant differences (*p* < 0.01) compared to the Erastin group. & or && indicates significant (*p* < 0.05) or highly significant differences (*p* < 0.01) compared to the OE-GPRC5D-AS1 group.

We investigated the impact of GPRC5D-AS1 overexpression on ferroptosis in HSKM. The full-length GPRC5D-AS1 sequence was synthesized and incorporated into the pCDH-CMV-MCS-EF1-copGFP-T2A-Puro plasmid, resulting in the successful construction of pCDH-GPRC5D-AS1 after enzyme digestion and sequencing. Transfection into HSKM significantly increased GPRC5D-AS1 expression, as indicated by qRT-PCR (Figure 4A). To assess ferroptosis, we exposed the GPRC5D-AS1-overexpressing cells to erastin (Erastin + OE-GPRC5D-AS1) and compared them to those transfected with an empty plasmid (Erastin). Two additional groups were included: one with empty plasmid without erastin (Blank) and another with pCDH-GPRC5D-AS1 without erastin (OE-GPRC5D-AS1). CCK-8 assays demonstrated that cell viability significantly decreased in the Erastin group compared to the Blank, while viability increased in the Erastin + OE-GPRC5D-AS1 group compared to the Erastin group (Figure 4B), suggesting that GPRC5D-AS1 overexpression reverses erastin-induced ferroptosis. To further evaluate ferroptosis, intracellular iron levels were measured. The Erastin group exhibited the highest iron content, while levels in the Erastin + OE-GPRC5D-AS1 group were similar to the Blank group (Figure 4C). Flow cytometry was used to measure lipid peroxidation with BODIPY Lipid, showing that erastin significantly increased lipid peroxidation in HSKM. Overexpression of GPRC5D-AS1 in the presence of erastin resulted in a notable decrease in lipid peroxidation levels (Figure 4D). MDA levels were also evaluated to assess oxidative stress (Figure 4E). MDA was significantly higher in the Erastin group compared to the Erastin + OE-GPRC5D-AS1 group, with the latter approaching levels seen in the Blank group. Additionally, SOD levels were measured to examine antioxidant capacity (Figure 3F). SOD levels were significantly lower in the Erastin group than in both the Blank and OE-GPRC5D-AS1 groups. However, SOD levels in the Erastin + OE-GPRC5D-AS1 group were higher than those in the Erastin group. These results indicate that erastin exposure markedly reduces antioxidant capacity, while GPRC5D-AS1 overexpression enhances the ability of cells to handle oxidative stress. Notably, this protective effect extends to the mitigation of skeletal muscle atrophy, as evidenced by Figure 3B where OE-GPRC5D-AS1 (with or without erastin co-treatment) significantly rescued Atrogin-1 and MuRF-1 protein expression levels compared to the erastin-only group (p < 0.05), restoring them to baseline levels comparable to the Blank group.

### GPRC5D-AS1 modulates ferroptosis through *SLC7A11* by targeting cellular lipid peroxidation

**FIGURE 5:**
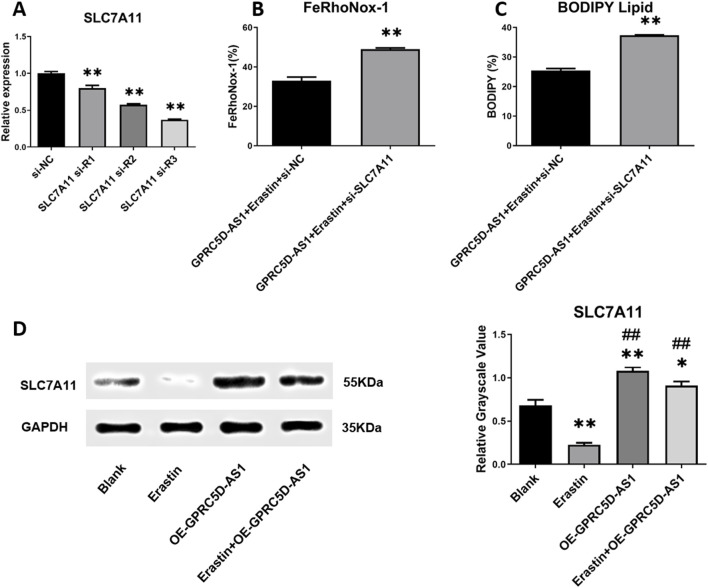
Detection of ferroptosis-related markers following SLC7A11 interference RNA introduction alongside GPRC5D-AS1 overexpression in HSKM cells. **(A)** Screening for effective SLC7A11 interference RNA, with * or ** indicating significant (*p* < 0.05) or highly significant differences (*p* < 0.01) compared to the si-NC group. **(B)** Intracellular iron levels in groups with and without SLC7A11 interference RNA alongside GPRC5D-AS1 overexpression. **(C)** Lipid peroxidation comparison between groups with and without SLC7A11 interference RNA under GPRC5D-AS1 overexpression, where * or ** denotes significant (*p* < 0.05) or highly significant differences (*p* < 0.01) compared to the GPRC5D-AS1 + Erastin + si-NC group. **(D)** Impact of GPRC5D-AS1 overexpression on SLC7A11 translation (*GAPDH* as loading control), with * or ** indicating significant (*p* < 0.05) or highly significant differences (*p* < 0.01) compared to the Blank group. # or ## denotes significant (*p* < 0.05) or highly significant differences (*p* < 0.01) relative to the Erastin group. & or && indicates significant (*p* < 0.05) or highly significant differences (*p* < 0.01) compared to the OE-GPRC5D-AS1 group.

To confirm whether GPRC5D-AS1 regulates lipid peroxidation via *SLC7A11*, a low-expression *SLC7A11* model was established (Figure 5A). qRT-PCR showed significant downregulation of *SLC7A11* after transfection with siR-1/2/3, with siR-3 chosen for further experiments. In HSKM overexpressing GPRC5D-AS1, erastin was added to induce ferroptosis. Flow cytometry revealed that *SLC7A11* knockdown significantly increased cellular iron content compared to the control group (Figure 5B). Additionally, lipid membrane peroxidation levels were markedly higher in the low-expression *SLC7A11* model (Figure 5C). These findings indicate that inhibiting *SLC7A11* expression negatively impacts the ability of GPRC5D-AS1 to reduce lipid peroxidation and mitigate ferroptosis in HSKM. As shown in Figure 3A, GPRC5D-AS1 overexpression significantly elevated SLC7A11 mRNA levels. Although erastin treatment attenuated this increase, residual SLC7A11 mRNA remained higher than baseline levels (*p* < 0.01 vs. Blank group), indicating that *SLC7A11* expression is transcriptionally regulated by GPRC5D-AS1. A parallel pattern was observed at the protein level, where SLC7A11 protein increased markedly after GPRC5D-AS1 overexpression. Following erastin treatment, SLC7A11 protein levels decreased but were still significantly elevated (Figures 5D). These findings confirm that GPRC5D-AS1 enhances both transcription and translation of the *SLC7A11* gene.

In summary, GPRC5D-AS1 reduces ferroptosis by elevating *SLC7A11* expression, thereby strengthening cellular antioxidant capacity and mitigating lipid peroxidation.

## Discussion

Our previous research indicated that the transcription level of GPRC5D-AS1 was significantly reduced in skeletal muscle tissue from older individuals (average age 79.33 ± 0.58) compared to healthy young samples (Zheng et al., 2021). Our study provides the evidence of GPRC5D-AS1/SLC7A11 dysregulation in human sarcopenia, the clinical sample size (n = 5 per group) and mixed-sex cohorts may limit generalizability. Notably, sex-specific differences in sarcopenia pathogenesis have been reported (de Jong et al., 2023). Future studies with larger, sex-stratified cohorts are needed to validate our findings. However, the consistency of our cellular data strengthens the mechanistic conclusions. This suggests a vital role for GPRC5D-AS1 in countering skeletal muscle ageing. In this study, we compared muscle samples from older patients with sarcopenia to healthy young controls, finding lower GPRC5D-AS1 expression in the sarcopenia specimens. *GPX4* and *ACSL4* are crucial markers in the study of cellular ferroptosis, with their significance validated in various studies (Kagan et al., 2017; Li et al., 2019; Yang et al., 2014; Park et al., 2019). Our analysis revealed significant differences in the mRNA and protein levels of *GPX4* and *ACSL4* in sarcopenia patients compared to controls. Coupled with other ferroptosis-related gene expression findings and tissue iron content measurements, these results suggest that ferroptosis may exacerbate the onset and progression of sarcopenia. Furthermore, literature supports *SLC7A11* as an inhibitory gene linked to ferroptosis, showing significant expression in related pathological processes (Dixon et al., 2012; Jiang et al., 2015; Du et al., 2021). Our findings demonstrate that the transcription level of *SLC7A11* were markedly lower in sarcopenia samples, reinforcing the role of ferroptosis in the pathophysiology of this condition.

*Atrogin-1* and *MuRF-1* are activated in skeletal muscle under specific stimuli or denervation, driving proteolysis via the 26S proteasome to induce skeletal muscle atrophy (Bodine et al., 2001; McFarlane et al., 2006; Gomes et al., 2001). As key mediators of myofibrillar protein degradation, these markers are widely utilized to assess sarcopenia (Gumucio and Mendias, 2013; Hong et al., 2019). In our ferroptosis model induced by erastin, both *Atrogin-1* and *MuRF-1* exhibited significant protein upregulation, providing mechanistic evidence that ferroptosis contributes to sarcopenia pathogenesis. After establishing ferroptosis model in HSKM cells via erastin, introduction of a GPRC5D-AS1 overexpression plasmid significantly enhanced cell viability. Assessments of iron content, BODIPY lipid staining, and measurements of MDA and SOD levels revealed significant changes in ferroptosis-related indicators following GPRC5D-AS1 overexpression. These results suggest that GPRC5D-AS1 effectively inhibits ferroptosis. Recent research has highlighted the role of non-coding RNAs in regulating ferroptosis. For instance, P53RRA enhances p53 protein retention in the nucleus, promoting ferroptosis and apoptosis as a tumor suppressor in cancer (Mao et al., 2018). H19 exacerbates oxidized low-density lipoprotein-induced injury to arterial endothelial cells via ferroptosis (Tang et al., 2024). PVT1, on the other hand, plays an important role in regulating ferroptosis during brain ischaemia/reperfusion by modulating the expression of TFR1 and p53 through miRNA-214 (Lu et al., 2020). In this study, we focused on the impact of GPRC5D-AS1 on ferroptosis within a cellular model of sarcopenia, but the role of *SLC7A11* in this process remains unclear. Before investigating their regulatory relationship, we predicted potential interaction sites, revealing that GPRC5D-AS1 can specifically bind to both the mRNA and protein of *SLC7A11*, indicating a possible mechanism for its inhibitory effect on ferroptosis.

The protein encoded by *SLC7A11* is integral to the system Xc^−^, which transports glutamate and cystine (Cancer Genome Atlas Research Network, 2012). Research indicates that *SLC7A11* mitigates lipid peroxidation by producing GSH, thus inhibiting ferroptosis in cells (Koppula et al., 2021). Iron overload and lipid peroxidation converge in ferroptosis, with ROS acting as a direct contributor to lipid peroxidation (Granger and Kvietys, 2015). Reduced glutathione serves as a potent intracellular antioxidant against ROS, linking *SLC7A11* activity to ferroptosis (Niu et al., 2021). In our experiments, *SLC7A11* was significantly downregulated in sarcopenia samples. Suppressing *SLC7A11* expression in cellular models led to marked reductions in cell viability, with concomitant increases in iron levels and lipid peroxidation. In GPRC5D-AS1 overexpression models, interference with *SLC7A11* reduced both the antioxidant capacity of GPRC5D-AS1 and its effect on ferroptosis. These findings suggest that GPRC5D-AS1 inhibits ferroptosis by upregulating *SLC7A11* expression.

In this study, we discovered that *SLC7A11* plays a crucial role in the cellular resistance to ferroptosis within the pathological process of sarcopenia. The occurrence of ferroptosis is closely related to intracellular iron load, levels of ROS, and the functionality of the antioxidant system. Our data indicate that the low expression of *SLC7A11* may lead to a reduction in the synthesis of GSH, thereby diminishing the cell’s capacity to combat oxidative stress and increasing the risks of lipid peroxidation and cell death. This finding is consistent with previous research. Moreover, we observed that the overexpression of GPRC5D-AS1 can upregulate the levels of *SLC7A11*, further enhancing the antioxidant capacity and viability of cells. This suggests that GPRC5D-AS1 may exert its biological functions by regulating the expression of *SLC7A11*, thereby influencing cellular sensitivity to ferroptosis. This mechanism provides a new perspective on the role of GPRC5D-AS1 in cellular protection.

The interaction between GPRC5D-AS1 and *SLC7A11* may extend beyond ferroptosis inhibition, potentially involving other cellular signalling pathways or transcriptional networks. Future research should investigate how GPRC5D-AS1 regulates *SLC7A11* expression and their interactions across various cell types and pathological conditions. Additionally, consideration of factors such as the cellular microenvironment, transcription factors, and epigenetic modifications that may influence *SLC7A11* expression is warranted.

## Conclusion

In this study, we systematically explored the relationship between GPRC5D-AS1 and *SLC7A11*, as well as their roles in HSKM and sarcopenia. Our key findings indicate that the overexpression of GPRC5D-AS1 significantly upregulates the expression levels of *SLC7A11*, thereby enhancing the antioxidant capacity and viability of HSKM. This mechanism suggests that GPRC5D-AS1 plays an important protective role in defending against oxidative stress and ferroptosis.

## Data Availability

The original contributions presented in the study are included in the article/Supplementary Material, further inquiries can be directed to the corresponding author.

## Abbreviations

GSH, Glutathione; ROS, reactive Oxygen species; SLC7A11, Solute carrier family 7 member 11; LncRNA, Long non-coding RNA; HSKM, Human Skeletal Muscle Myoblasts; PBS, Phosphate-buffered saline; qRT-PCR, Quantitative real-time PCR; WB, Western blot; SOD, Superoxide dismutase; MDA, Malondialdehyde.
