# Supplementary material for: Regulation of SLC7A11 by LncRNA GPRC5D-AS1 mediates ferroptosis in skeletal muscle: Mechanistic exploration of sarcopenia
Source: Front Mol Biosci. 2025 Apr 16;12:1557218. doi: 10.3389/fmolb.2025.1557218 (PMC12040812; doi:10.3389/fmolb.2025.1557218)
Supplement: Supplementary file 1 [file Supplementaryfile1.pdf]

Supplementary Material

Table S1. Clinical patient sample demographics.

Sample	Gender	Age(year)	Heigth(cm)	Weight(kg)
Control 1	Male	17	174	56
Control 2	Female	37	152	60
Control 3	Female	30	160	53
Control 4	Male	39	175	80
Control 5	Male	23	179	64
Sarcopenia 1	Female	80	150	44
Sarcopenia 2	Male	79	175	80
Sarcopenia 3	Female	70	165	55
Sarcopenia 4	Female	79	150	60.6
Sarcopenia 5	Female	81	160	70

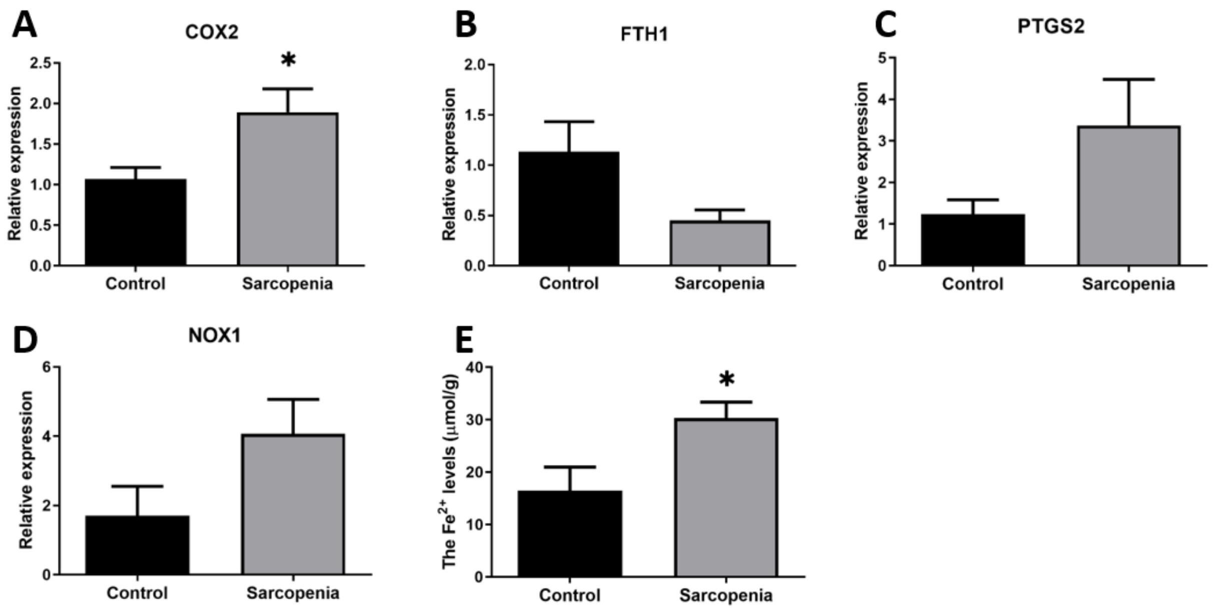

Figure S1. Relative expression levels of target genes in skeletal muscle samples assessed by qRT-PCR, along with differences in iron content between two sample groups. A-D The relative expression levels of target genes, while panel **E** illustrates the iron content disparity between skeletal muscle samples from sarcopenia patients and healthy controls. * or ** indicate significant ($p < 0.05$) or highly significant differences ($p < 0.01$) compared to the Blank group.

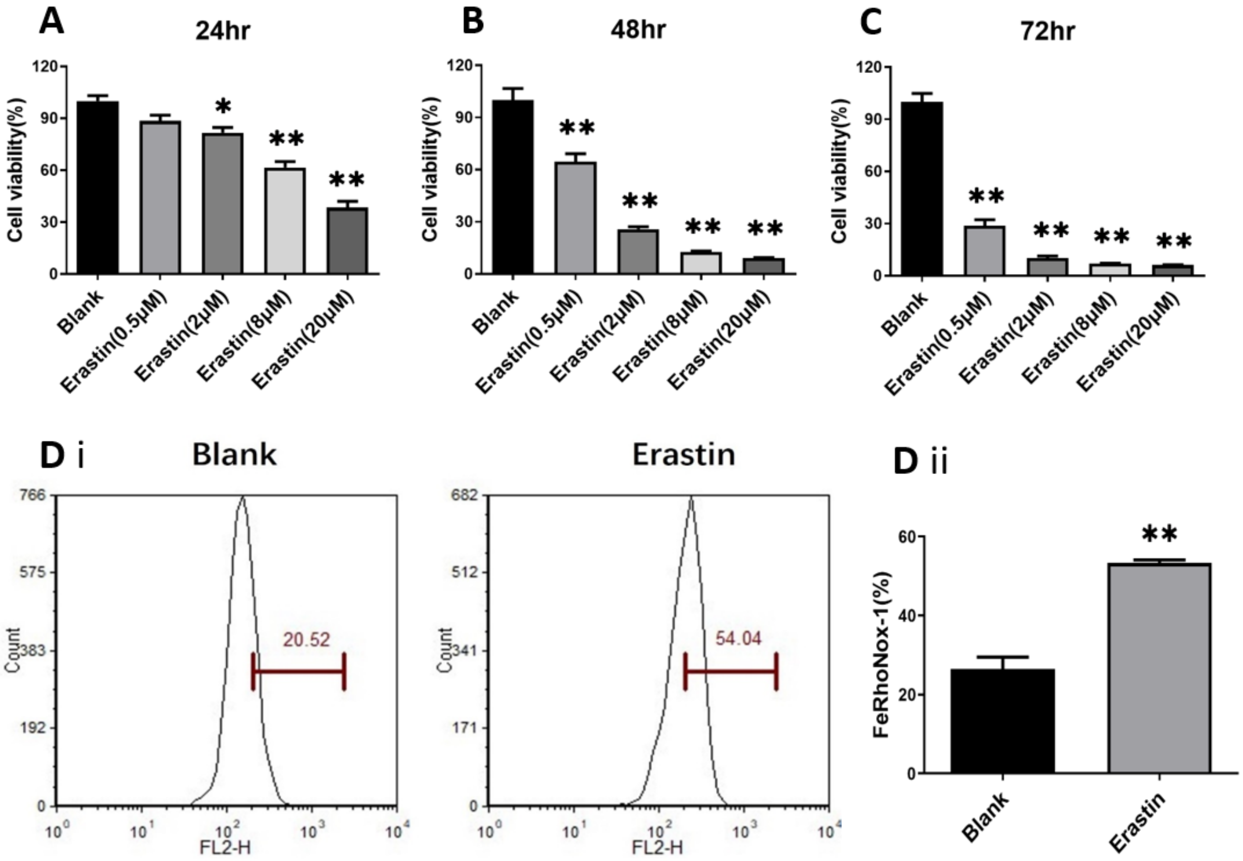

Figure S2. Establishment of an Erastin-induced ferroptosis model in HSKM cells. A-C Cell viability of HSKM cells exposed to varying Erastin concentrations for 24, 48, and 72 hours, respectively. **D** FeRhoNox-1 assay results indicating intracellular iron levels. * or ** indicate significant ($p < 0.05$) or highly significant differences ($p < 0.01$) compared to the Blank group.

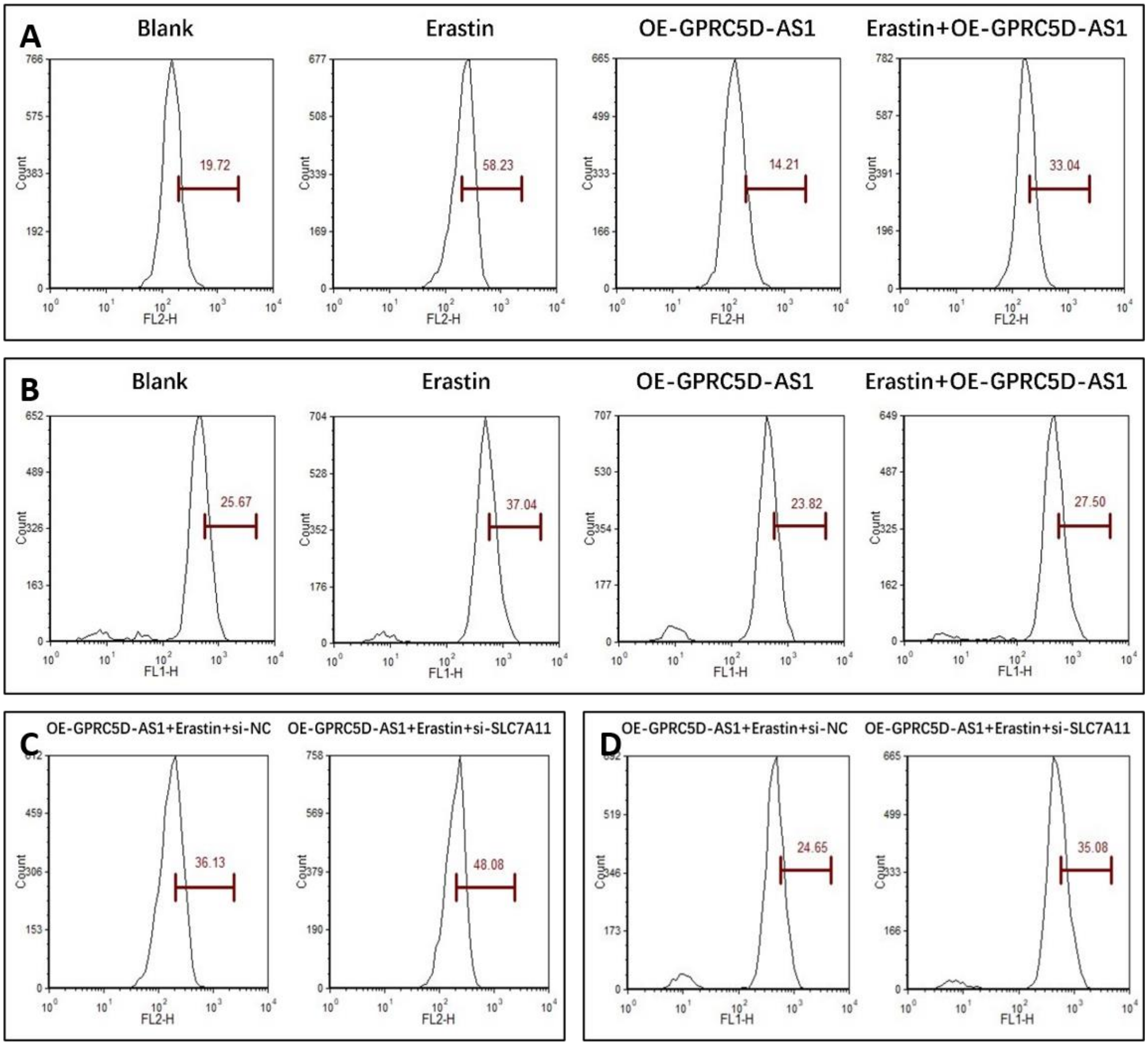

Figure S3. Consolidated histogram of flow cytometry analyses of ferroptosis-associated biomarkers. A Iron levels corresponding to Figure 4C. **B** Membrane lipid peroxidation levels corresponding to Figure 4D. **C** Iron levels corresponding to Figure 5B. **D** Membrane lipid peroxidation levels corresponding to Figure 5C.

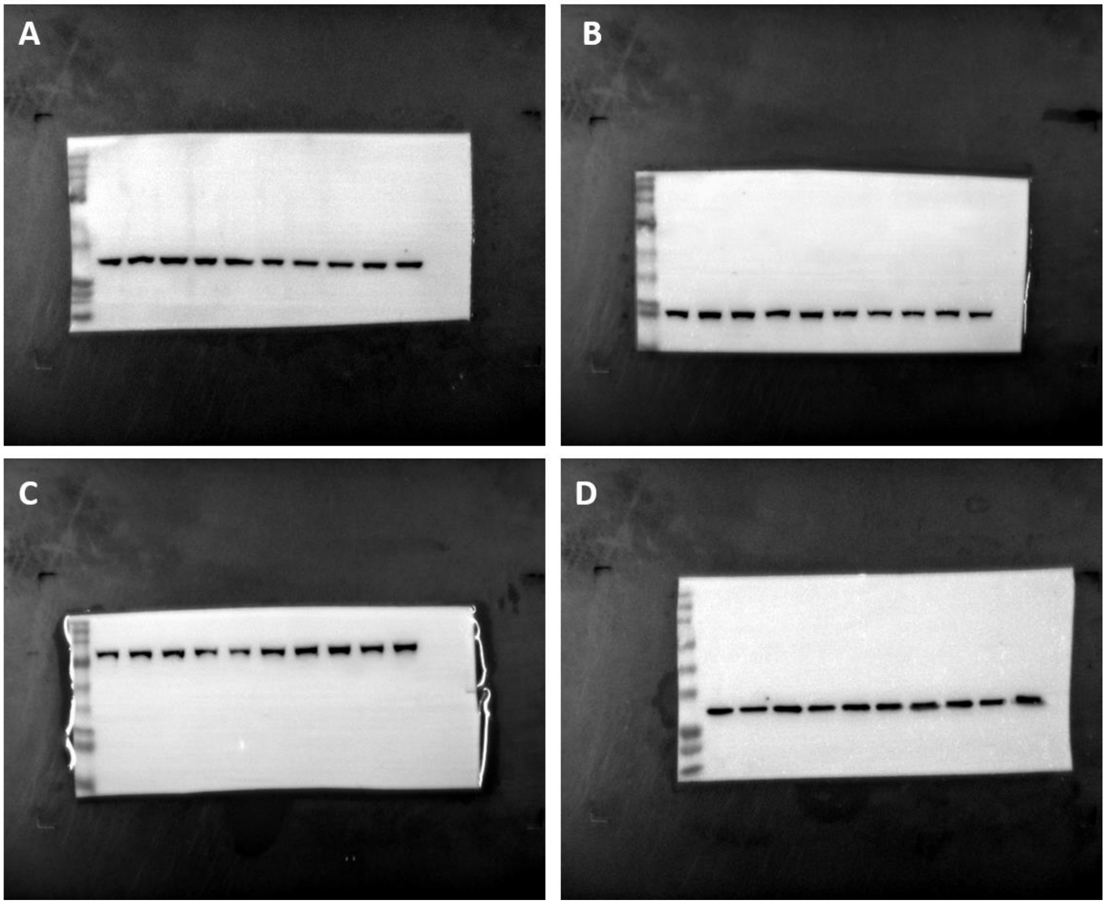

Figure S4. Full Western Blot membrane images of three target genes (*SLC7A11*, *GPX4*, *ACSL4*) in skeletal muscle samples from clinical patients, using *GAPDH* as an internal control. A: *SLC7A11*. B: *GPX4*. C: *ACSL4*. D: *GAPDH*.

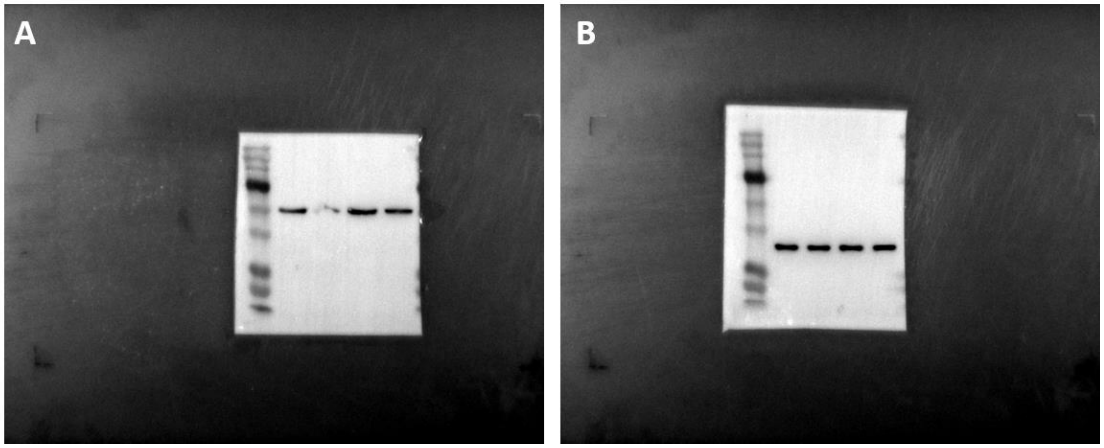

Figure S5. Full Western Blot membrane images of *SLC7A11* in HSKM, using *GAPDH* as an internal control. A: *SLC7A11*. B: *GAPDH*.

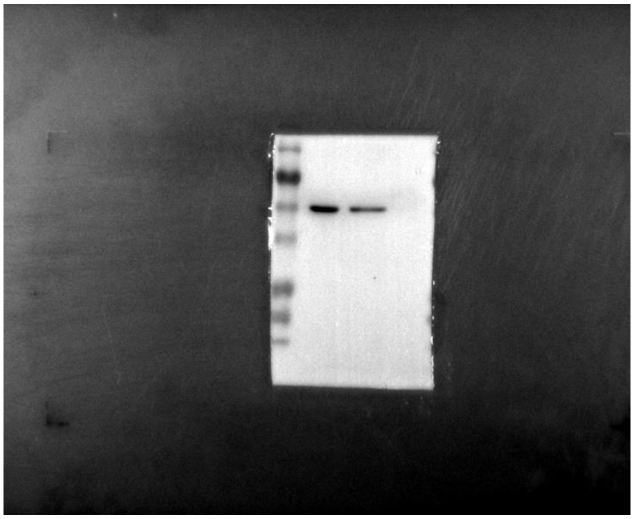

Figure S6. Full Western Blot membrane images of RNA Pull-Down Analysis.

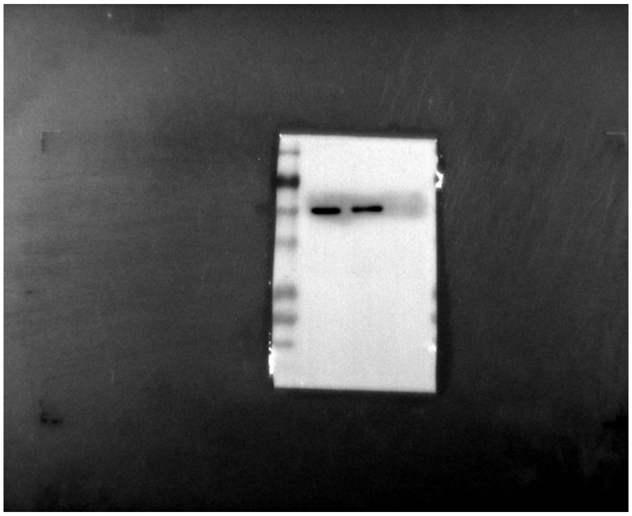

Figure S7. Full Western Blot membrane images of RIP.
