# Supplementary material for: Regulation of SLC7A11 by LncRNA GPRC5D-AS1 mediates ferroptosis in skeletal muscle: Mechanistic exploration of sarcopenia
Source: Front Mol Biosci. 2025 Apr 16;12:1557218. doi: 10.3389/fmolb.2025.1557218 (PMC12040812; doi:10.3389/fmolb.2025.1557218)
Supplement: Supplementary file 2 [file DataSheet1.pdf]

1. **Prediction of the Interaction Between LncRNA GPRC5D-AS1 and SLC7A11 mRNA via LncTar (http://www.cuilab.cn/lnctar).**

Query	Length Query	Target	Length Target	dG	ndG	Start_Position Query	End_Position Query	Start_Position Target	End_Position Target
GPRC5D-AS1	1743	SLC7A11	9645	-38.17	-0.0223	1	1743	1056	2798

5'AAACATACACACACCCCTGATTCCAGAAACACAACAGGAGCTGACTCAAAAGGGAGTGTCTG
GTGCAGGCATGCAGGGAGGAAGAATGACTCTGAGCTTGACCATTCAACTTCGAAACTTTAACAT
CAATGGATCAGCACACACTGGTGTACGGTCTGCTATGCATGCAGCCCTGTGCTAGGCAAAAGA
AGTATCAGACCAGGAACTGACAGCCTCACTGCAACAGGAAGAGAGGCTTCACACAATAAGCGT
TTCATTCAACCAGGCTGGACCTGTGCTTATTGAGTTGAACATCACTGAGCACAAAATGCAAGCC
AGACACCATAAGAGCTGTAAGGATGATAAAGATGTCTTTCTACCCTCGAGAGCTCTAAGTGTGG
TCAGGAGAGAAACACATGCAAAGATTATGCTGGCTGGAAGGAAATGCTGGAATGAAGGTGGAA
GCATTCTATTCATCTAAAAGCACCGGTAATTAACGATGCTAATAAATGTATAATCCCTTTGTAAA
CATCTCTTCACAAGGTCATAAAGAAGGAAGACTGAATGAAGGACGCCAGGCTTTCATTTTAGAA
TTAATTTTGTCATTGAATGCACTCACTGCATTATTTGATAGAGTACTTTTGCATTTGAAGTAGTAC
ATTTGCCTTCTACCATCTCATTAACATTGCTTTCAACAGAATCAGAAATAACACAAACCACTGCA
GGGTAATTTTGAATGCTCATTTGACTATGCAGTGACATCAAGTGGCGAGTTGAGGAATGATCAT
ATGCTGTGTGAGAACTCCGTGTTAAAAATTCCATGGAGCCAGGCTAGACACCACATTTGGCACT
TTCAGCTGTTTAGATTTCAGAGAATTAAAAGGAAATGGTCACCGACCTGCCTTTGATAGTACCCG
TATACCCCTTCCCACCATAAGAAACCAGCGTTCCTTAGAGAAATGGCTAATTCTAGCTCTAGGG
CAAGAAATGCTAAGATGTGCTCAAAACATCTCATTATACCAGCAAGCCAGGGAGCCATCAACG
ACTACTGGAGCTGTGTCAAAAGGACCAACAAACCAACTTGAAGAGGCTCCCGCTGGCCAACAG
TGGGGCAATTAGAGCATCAATAAGAATAATATCTGTAACAAACTGAAACGTATCAAATATGTTTA
AATTCATATGTTTATAATTACACTCAAAAAACGAAGATGCAGACCACTTTACCTTGAGGATGTTA
CAGAATGAAATTATCATTCTGAAAACTGGTAAATAAAGAGAAGGTATCAAGTAAGCATGTATCT
GGCTTTTTCTATATGAAATAAAGGAAGCCAAATAGTAAACGAGGGGAAGTTTCTCTTTACTGAAA
TATTGCAGGTAATAAAAGAAGAAGGAGAAACAGAGAACATTATCGTTTTATGGCCTCTAATGGA
ACAAGACATCTAGGCAATGATGAACTATGGTCACCAACATCACTCAATCAGGGACAATCAGAC
ACTGTGGGCCCCTGAAGGAAGTAAACACCACCACGTAGGAAATATTCTCACCAAAACTTCAAA
AACTTGAATCTGATCTAGTCTTTAGTTTCAACTACTAATTTACAGGCAATACGGGGCTCAGAGGG
ATGTGGTAAAATGTCACGGAAATATAATCAGCAAAATCCAGTACAGATAATAAGAAGACAAATG
ATCCACAGATGGAAAGGAAGAATGTAAAGTTGTGTTGGTAAAGAAATTCACAGATTAATAAAAG
CTTAAAAATGTACCA 3'

|||.......|||...||.....................||.....|||..................||.|||.......................||....||.............|||.................||...............................||...
............||............||...............||................................||.........||...........||............................||..||...||...................|||..||.........
.........|||..|||.||.........................||................||......|||........|||......||.........||..........||...................||.............||.......|||..............
..................................||......||....||.....||.......|||.....||.........||.......................................||.........................||.||......................
....||..||...................||.............||||.....................................||...
....||||...|||.............................||...|||.....................||................||..................||
....................||.............||...||..||.........||.....||...|||.........||||.......||.......||.......||...........||...........................||..||.....................
.||.|||...|||.||.||..........|||........||.........||......||..........||......................||.||.................||..
|||............|||..............||...........||...................................|||...........................|||.....||.......|||..||

....|||...................||.............||.||.......................||.||....||.....................||||..............................|
3'
AATACATTTGATAGTTTACAAATAAATTTAAAGGTAAATTTTATAAAAGTTCATTTTATACATGTTT
TTACCAATATTTTACCAACTTCGTTGATCTTCGCACTGTCCATATTATGTATATTTATGTTGGTTTT
AAGTTAAGTTACGTTTCAACTTACTGTAGTATAACGTGGTTTTAAATAAGGTATGTTTTCGTGTAC
GTAGTTCTCAAAGGTATTCTACTTTTGTTTGTGTGAATGAAGTATCGTAGAATGGTGAATGAATGT
GTTTATCGGGTATTTGTGGTAGACCGTAACACTAACGTCATGGTCTTGAGAGGGGTCTCCCTTGA
GTAAATCGATATCTTCTTAGGTAAAATAAAGTGTATAGTGTACGAACACGTCCGTAGTCACGATC
CTTGGGATTCTTTGTTGCGTTAGGTGTCTACTTTCAGAGAGACGTGGTAAATATAGAAGTATCTA
TTTATAGAATCAAGATTATACTAACCTTACACCTACGTCTTTATTTTACGTCAAAACGAGAAATTC
TTAAAATAGTTACATTCTGTAACATAATTTAAACATATTTTATGTGTGTTAGGGGAGATGATTCAA
AGTACTAGTGTCACGGTCTCACTTCTTTGAGACGAACTCAACTCCTGGTCAATCAATCCTATAAC
TCCTAGTGAGAAAAAGTCGACCTAGAGAGACAAGAACACAAAAGTTAGTGATCTGGTTTGGGT
GCTACGGGGACTTTAAGACCCTTACCATGTTCCAGTATTGATTACGTAAGGTCTAGGTTCAATCC
CTAAATCGACCAGATATTTAATGTCAAATTCGTGTCTAGTTTCTCTACCGACGGTCGTATAGGAA
GTTCCCATTGGACTCAAGGTTCTGAAAGGGTGACCCGATTTACCTGAGGTGAACAACGTTTAAT
ATTTACGAACAGTGTCTTATACTTTTTATAAATTTATGTAAGTATCTATCATGGATTATTATACAAT
TGTATTTGTATTGTTGTGTGTATAATAAAAAGATGGGGAACCGTTGACTTTTACTTCAATGGTAAG
GATCCGGTTTAAAAATCTGTTTCGAAAGATTTTGGTAGAAATATTTCATTTAAGTCTATACGAATG
TTATTTTTCTGTATTTTCTAAGTAGGACTCTACTTAAGACTCAGTTATTGATTTTTGGTAAAGATGG
TCACGTAGTGATGGTACATTAGGTAAGATGCGTTCGAGATGTTTATAACTCAGTTTAGGACAGAC
AGTCTTTTACTTCTGGGTTATTCAAACGGCTTCATAAGTCATGTTCTCCGGTAAACAGAACGGAA
AAAGACTGTACACGTATGATATTTTAGTGTCCATCGGTTGTAAATCATAGTCATTTTTTGTTGATG
CAAACAAGTGGACAAACCGTATCCCTCTTTTGTTACATAGAGTATCGTAATTTACTATGTCGGAA
TTGTGTATACTACGAGTATAAACGTTTCAAGGGTTTACAACTCTTCAAGATCACTTTTCAGTATGA
TAACACGTTTCTACTTTTAAACCTCGGTTACAGACATAAGTTTTATTGGTTTTATATAAAATTTCGT
TTTATATAGGACTATGATGATATCTAAGATCCTTAACAGGATTTTCTCATTTCACAACAAAGGAA
AGACTTGTACTTATTGTAGTTTCCTTCTTGGGTCAAGAATTCTGAATTCATCCTTTAAATATCTTTA
AACTAAATATGGTCATCATTATTGTAAGTATTCCTTTTTGATAATCCATTGTTAAAAGAGGTTCTTC
TCCTAGTCTAATGAATTTTAACAACCTCTTAAGACCAACAAACACGTTATTAGTATCACTAAATGT
AACGAAAAGAAGAAAGTCTCGTTATTCTTTCAATAAACCCCATCATGAACTTTATTAAGTATATG
TGTCAGTAAATTGTATCTAGCAAAGATCAGCTCATACTCGTATGTAAGTACCCTTGTTAGACAAA
GTAAGAAGTTTCATACAAACATGAAACCTTACTTCCTCTTTAAGATCCCTTTTTAATTAAGTCTTT
AGAAAACTTTATTCTGTCAAGTTTGGATCCTGGTCCATTGGTGTAACGTAACGGAGGGTCACACT
ATACTGACACGAAGGTTCATACGTAGATTATTTCAATCAATCATCCTTTTAAACTACAGATTTAAA
ATATTATGTGTAAATTCAGTCTGGTGAGTGTTGGGTTGTTTAATATGTCGAAATTGTACGTTTCCG
GATTATCCAAAAGGGTGTTCTACGACATTATTATGGGACAGTGTGTTCCATGAGTTTTATGTCAG
TGGTGACGTTTTCTCTATGGACACGTAATGAGAAAAAAAGGGGAGACCGAAAGTCGTGCCGGA
AGACTCCGAAATTGTTCTCCATTATTCCTGAATCGTTCGTCAAGAGGATTGGTTATGTAATTAAGT
GAACTTTCAGTATGAAAATTTTTTTTAGTCATCACATATACAACTCCTTTTTTTCGTACAAGTGAG
ACCTTGTTTCAACCGGGAGATACCGAGAGTCTACGGATTACGAGATGTCAAAACACTCTCTTAA
CAATTGACATTTCGACATTTCCGCCAATAACCATGATATTTACATGTGAACTAAGTTCATGCGAC
TTTGTGTGTGTACATTGTCGATAATCCTGTGACATTGGCAGGAGACACATAAAATTCCGGTTATC
ATTGTCTCTCCTCCCTTTTTTTATTGATGGTTCTTTTATTTTGATTACCCATCTAAGTGTGATTCTTTT

AGATCCATTAATATATTTAAGTAACAATACTCCAACATCTTTCCTGTCAATAGAGTGCTGTATACC
TTTTAACCACTTAAAGGACACTCTGATACTAAATACGAAAACACTTACATATGGACTAGATTTTA
ATCTGTTTTGTAAATAAAAATCTGTGGTTGATTAACCTTTTATATTCCTTTCTTTATTAATAGAGGG
TTTTAACTGATAATTTTTTATTAAAAATTCATGTCATGATTATTTGATTCCTACTGGATCTCTCGTAT
GGAAATCGATAATAAATTTTTAGAAAAAAAAAAAAAAAAAAAAAAAAAAACTCTACTTCAGAGCGC
GAGAACAGGGGATCCGACCTCACGTTACTACGCTAGAACCGATTGACGTCGAAGACGGGGAAC
CCAAGTTCACTAAGAGGACGGAGTCGGAGGGCTCATCGACCCTAATGTCCGCGGACGGTGGTG
CGGGCCGATTAAAAATATAAAAATCAACTCTGTCCCAAAGTGGTACAACCGGTCCGACTAGAG
CTTGAGGACTGGAGTCCACTAAGCGGGCGGAGTCGGAGGGTTTCACGACCCTAATGTCCGTAC
TCGGTGGCACGGCCTGGATAAATTTTTAGAAAAACTTCATGTCATGATTATTTGATTCCTGATGG
ATCTCTAGTGTGAAAATCTATAATAGATAAAATTGTATCTAATTTTTATGACAAATATACTTTTAAT
TCGAATTTATGTGCATATCCATTATTAATAAAACGGGTATATGTTCATTACATTTGTCTCCGATAG
TTCCACTGGGTAATAGATGAGATTAAATATAGGTGATACTCGAAGAAGGGTCAACAATATGTTA
CGGTATAAAATTAAGGACTGACTTACCAATCCTTTCGTTTTACATTGAGACATTTGAACTAGTCGT
GATTACATGTTGTATAATACCTCCCATTTCACTTCGTTCTTATATAAGTTCATTTTATTTTACAGAA
AGTACCCGTAAGTTTAGGGTGTAGAGAGATATAAAGAAAGGGTTGATTCTCGATATTAATTTTAG
GGTAAATAATAAAAAGATATCAAGTCTTAGTTGCATATTTTATACTACTTTCACTTTGATAGAGGG
ATATATAGTGTAGTATATATAACACTCTTTCAGTGAACAATATATACCTATAAAGTTAATTGAGTA
TTTAAAAAATTTTTGTATAGGAGGTATGAATAAAGATTGTCTTTTTTTATTGTCCTTAATAAAATGAA
ATGAGTGTACCTGTTACGTCTAAAACACCCCCATCTTCACATGTGTTGAAACGACCAGAAGAAG
TTGTTTTAATCATGTCTTAAGGATTATGTTTCAATCCAAGTCCTGGAGCTTACCTTGTACAGATTC
ACTCTCCGTGACTTCGAATCCAAAGTAATCGAAGGACGCTCTAGGTGGATACGTGTCGTTTGTT
GATTAAAAATTATAAAGAGACTGATATAACGTATTGTAATTTTATAACAATGGAAGTCAGGTATG
ATAGAAAGTAAAGGACTAAGAGACCGGAAGCCAAAAAATAGAAAAGTATGGAATATGGAGTTA
ATGTTTTCTAGTTGAAATTGTTTCCAGTCTCAACGAGGGAAAGAATCGTCATATAAGTAGACGTT
TAGTTCTATTTTATTACTAAAAGGACATCTGACTAATGTCAAGTACTCCTTAAGACAATACATTAA
TATGAGAACAATTGGGAGATAACTATACAGTTTAACATTAACCCCTTTGTTAAAATGGTCTGTGT
ATTCTGCTTGTCCTTTAGACAAAGTTGCACTTATAGAATCACAACCTCTTTGGCAGTACGTAACG
ATTGCATTGGATTATTCGACAGGTTTAAAACACAGGTGTCCCGACTCCTCGATGTCAGATTTACG
TATGCGTACAACCCTTGGCTTCAGTCGGTAGAAGGGATCCTGTTTAGAAATGAGAAGATTTCTTC
CTGTTCTTATTCCAGATGAAAGTCGATTGGTAAATATTCCAAGTCAAAAGTAACCTAGTTTCCTTA
TATTAGATTATGAAAAATTTACCCCAGTTTTGTCATAATATTCCTTTTATAAATGACGTTTATAAAT
ATTAACACATTCCTCTTAGTAAGAAAAAATTTTAGTTCAACACAAAATCGAAATCTTAGAAAATTTT
AGTGTTCATAAACTCTTAGGTAGGGGTGTTTTTAATTTTTTATGATATAATCAATAAAATTTGTCAT
AAGTAATGAAAATGACAAATACAATATTATTTGAATACATATATTTGTAAACTTATACGTACCATG
TTTCATGTTCATGTCTTGTACATCATTCATCATACACGTACATACACACACACACGTATAGCATAT
TTTTATACTCTTTTTAAAATGAATATATTTTTGTATATATATTTATTCACAAGTCCTCTCAAAAACTC
TTAGACGAAATGTATGACGTTAAAACTCACTTTTAATACCTATAATTAAAGGACTAATTTCCTTGA
CACAGAAGAGTACTACTCAGGATTTTTATTCGGTTTTATGATGAATTAGAGGTAGTGAAAAAAAG
AATTAATGACCGACTGAAAAAAAGACGCACTTTACCGTAATGTTACCGTCCCTTTAAATTATTAC
GTTTTTAGTGAAAACTTTTACTCTTTCTTATTAAGATCAATGGAAAATTTAACTATGAAAATTTCTC
TTTAAGTTTAGTTTTTATTTATTTAATTTCAGACTCTGGTTAAACGGTGACACTTATATTCGTGTAA
TTGGGGTCCTTCTCGGTTCTTGATGTGTTTGGAGAGATACTCTTAAATGGTCAGAAGAAAGTAAA
CCGTTCTTTTTCGAGTCCTTTTAAACGAACAAATTTAAGATACTCGGATCAGATACCCCTTTTTTTT

TTATCCTTATACTTTCTTTAAGAATGGAGGTCGTTGTATAGTATCCCTCTCTACACCTGTTTACTTG
TTATAAATGACCTTTATCCTTAATATTTAGTTTAAAACTTTTGGGATACGATTCCTTCCTTCCTCAA
TAATATTTAAGTGAGTCTCTGGGTTTGCAATCCAAGTCGATTTCTTTACAGTCAGAACCTATATAT
AATTTACAAAAGTTCTCGTTTTAACTTTAATGACTTAACCTTTACGAGGTTTAATATCCAGTAGTA
AAGATGAAACCTGTTAAAAAATACTTAAGATACAAACGGAAATTATTAAAATTAACCATATTAAG
AAAGTTTTTAGGAATAAAAGTTTACCTGTTTTTAATTTCCTACTTTGTATGAGACCGGTACTGATT
GGTAAAATGGAATCTACAAATATTTATTGTCAAGTTTTAAAGTAGTAATATAATGAGAGTAAACA
TTCAAGGTGATATATATATATATATATATATATATTTTTCACACACACACACACACATATATATATAT
ATATATATATGTGTGTGTGTGTGTGTGTGTGTATGTGTGTGTATATATATATATATATTAGAAAAA
AAAAACCTCTACCTCAGACTGAAACAGTGGGTCCGACCTCATGTCACCACGTTAGAACCGAGTG
ACGTTGGAGGCGGAGGGCCCAAGTTCGTTAAGAGGACGGAGTCGGAGGAGTTATCGACTCTAA
TGGTCGTACACGGTGGTACGGGTCGATTAAAACATAAAAATCATCTCTACCCCAAAGAGGTACA
ACCAGTCCGACCAGATCTTGAGGGCTGGAGTCCACTAGGTGGGGGAGTCGGAGGGTTTCACGA
CCCTAATGTCTACATTCGGTGGTACGGGCCGAGTGTCATAAAAGATTATGACCGTTTTGTTGTAG
AAGACTTGTGTTTGTGAATAGGTGTCTAAGAGTAAAGTTAAAAGAAGAATAGTCAACAGAGAAA
ACGTTCAACTTACTATTAATTCCACACCTTTAATGACTGTTTCGGATCTATATCTTCACCTTTAAAA
GATCTTTGTAGTCAACATTTAGAATAAATGTCGCTGTAAAGAAAACGTTAAAAATACAATTATAT
ACGGAAAAGGATTAATTAGACCTGTCAAAGTAGACTTCTCTATAAAATTGTCTTCTTATATTTTAC
ATATTTCTTTTACGTGTTTGACAGTTTTAAGTAGTAGCACATTTTTATCGGTATTTAAATTTTTTGAA
GTCTTAATGATTGTAACGTACATCTATACTAATGTCTATACCCTGATAATAAAACTTGTAGAAGA
AACAACCACTAAAGAGAGTACAACTATTACATTAAACTTCCGAATTTTACGTATACAAATCTATC
TGAGTAGTCCCCATTCAATAATCACTTTGTCCACCAACAAGATTACTCCTAAATTATGGTTTGATT
GAGAACGAGATTCTACACAATATGTGAAAGGACTAGTTTGACTACACTTTTTTTTTTTACTGTCTA
TTGATTTTGAAGGACTATTACTTACTTTTTTGTAGACTGTATTATTTAAGAGTTCCTAAAAATGAAT
TTGTATTGAGGACCTACACAGAGTATTTGTCATCGGGGATCTTTGAGAAGAAATTACATAAGTTG
TTAGGGGTGACTGGAAACAAGTTTCACGAGACCCATATTAGGTAATTTCGTACTCACAAACAAT
AGATTTAACCATAGATTCCCCTTCGTTACGTTACAGTTACATTTTATTTAAAGAAAGAGAGGAGA
AACGGTATTAGTACTACATACGAAAAAAAGTCTTAACTTTTGTGAAAAGTGTCATATCTGTAGAT
ATCACACATTTTGGTAGAAATGTTTTGGGTATATATGTCTTTTTAGAGTCAGAAGTTAAACTTCCA
AGAAACGGCACGATTGTATACAACATCTCTTACTGGTGTATCATTAACAGATCAGAAAAGAGGA
TTGGGGTTATCCATCCATAGTCTCTGAGTTCTTAACACGCTGAGTATCTTATTGACGTATAAGTGA
AAGATATTGATGTATCTCAATATTGATTTAAAGAATCTTTAATCAAGCTTATACGATTTTATATACT
TATTTTTATTGACTGAGGAAAACAAATAGTGGTTTCAACATTAAGAGATCTGAAAGTCTTTTACTT
CATTTTTAGGGATAAAACACAGAGGGGAACCCGTCTAACGGTTCTAGAGTTCAGGTAATCAAGT
ATTGAATAGAAGAAGACCATGTTGAAGGTCATAATAAACATTACAAGACCAATAAAAGAGGCT
GTAATAAGATTTGGTGGACCCAAAGAACAGGGTATATTATTTCTCTATTATGCGTCCCTGAGGTC
AGTCTCACTACTGCTTCGGTTAGGGACATGATTTACCCAGGCTTATCTCCCTTTCCCGTTGGTACT
TCTCCGTACACTTCCTTTTGTTTCGACCCTACTTGTCACCGTGGAACTTTCCTGCTACGTATAGAC
CCGTAAACATAGCTTCTATTTAGTCGGGTCGTTGACGGTCGGGTTATTTTTCGGTGGACCGTTTT
GACTCCTTTAAGTTTTCTGACAGCTCCAGAGGTCTCTTCTCGTAATAGTAACAGTTTCCCACGTTT
TGTTATTGTCGACCATCTCCTCACACGAACGCCTGTACTTAGTACCTCTCCTAAAGACCTTCCAC
TGGGAGAGCTCTGCGTTGTATCTTATTGGACCTCTGTCGTTTGTGTGGTGGCAAGTACCTCGGTT
TCGTCCTCTCCCGTTGTTTCTAGCCTTGACGATTACTCTTTAAAGGGTCATCGGCGAGTCTTTTCC
AGTGACGGTGACGTAAACTTTCGTCGTCGAGGAGTCGTAATTACCAGCATTTCATCCGGTGTAA

ACAGTCGTGTATCGGTTACCACTGTTACCGGTACCTATATGTATAACGTTCCCCTTACCAAAAAA
GTCCCAAAAGATGAAGAAGTCATTGTTTCAACTCCATTTTGGTCGGTCGTATACGTATGTAAGGT
ATTATTTTTCGGTCACCGTTGGCGCATTATGAACTTAGAGAAGGACTTTTCCGCAGAAATTTCAA
GACGCAAACTGGAAATTAATCGACGTATTGAGGTCCCTGATATTAATAGTCTTAACGACACTCG
AACGTTTTCCAATTCTTTTAGACCTAGGCCCGCGAGGTCGACTGTGAGTACGATAAATCCTGGTA
GTGATGTCAATACGGGTGTCGACATTACTCGAACTAGCGTTCAAGTCCCTAAAGTGTAACTTATT
TTTTACCAAGGTCTTACATCGCAGGTTTACGGTCCCTATAGTGTCGTCATCGACGTCCCGCATAA
TACTCCTCAAGGTGGGTCTGAGCATGTTTTCGACCATTACCTGGTTTCTGAAGGTTTTATATACAC
ATTACTGGAGGTCTAAAGAAATATCAACAAGGGTTAAGTCGTATTCTGTTTCGAGGTTTATCACT
GTCCTGGGGTGTGTGGCAGGTCTACCAGTCTCTGTACGGGTGCGACGGGCACAAGACCTCGTG
CGGGAATCCTCTCTACTTCTAAGGACGAGGTTACTACCACGGTTACTATTACCTCTGAGGGGAG
TCATTTCACTGAAAGGAGAAGTCGACGTGAAAGAGGACGGGTCCACCGAGGAACAACGGGTC
CCTTCCGTCGGAGGGCAATTGTAAAGGGACGTCCATTGGAGGAAACCTCTACCACCTGTGTTGT
CCGAAAGACTGGTATCATCCCTGTGTGCCCCCTTTTTATTTTGTCTCCCTTTCTTTTGTTTTGAAAG
TTGAAACCACAGAGAACCACAATGACTAGCTACAGAAGGAGACGAAAGTCTGACAGAGAGAG
TCGCGATATCACAAGTGTCCACTTTTGAGTTTCCACACGAAAAAGGAAGTGTCGCTAGATTAAT
GATGAGTCTTTGTGGACACATACGTAGCACGAGAGTTAAGAGGTGGAGGAGCAAGGTGGTGAC
GACGACGACGACGACGACGGCGGGATAGTAATGTTTGG 5'

2. **Prediction of the Interaction Between LncRNA GPRC5D-AS1 and SLC7A11 Protein via RPISeq (http://pridb.gdcb.iastate.edu/RPISeq/links.php).**

Protein:

MVRKPVVSTISKGGYLQGNVNGRLPSLGNKEPPGQEKVQLKRKVTLLRGVSIIIGTIIGA
GIFISPKGVLQNTGSVGMSLTIWTVCGVLSLFGALSYAELGTTIKKSGGHYTYILEVFGP
LPAFVRVWVELLIIRPAATAVISLAFGRYILEPFFIQCEIPELAIKLITAVGITVVMVLN
SMSVSWSARIQIFLTFCKLTAILIIIVPGVMQLIKGQTQNFKDAFSGRDSSITRLPLAFY
YGMYAYAGWFYLNFVTEEVENPEKTIPLAICISMAIVTIGYVLTNVAYFTTINAEELLLS
NAVAVTFSERLLGNFSLAVPIFVALSCFGSMNGGVFAVSRLFYVASREGHLPEILSMIHV
RKHTPLPAVIVLHPLTMIMLFSGDLDSLLNFLSFARWLFIGLAVAGLIYLRYKCPDMHRP
FKVPLFIPALFSFTCLFMVALSLYSDPFSTGIGFVITLTGVPAYYLFIIWDKKPRWFRIM
SEKITRTLQIILEVVPEEDKL

RNA:

AAACAUACACACACCCCUGAUUCCAGAAACACAACAGGAGCUGACUCAAAAGGGAGUGUC
UGGUGCAGGCAUGCAGGGAGGAAGAAUGACUCUGAGCUUGACCAUUCAACUUCGAAACUU
UAACAUCAAUGGAUCAGCACACACUGGUGUACGGUCUGCUAUGCAUGCAGCCCUGUGCUA
GGCAAAAGAAGUAUCAGACCAGGAACUGACAGCCUCACUGCAACAGGAAGAGAGGCUUCA
CACAAUAAGCGUUUCAUUCAACCAGGCUGGACCUGUGCUUAUUGAGUUGAACAUCACUGA
GCACAAAAUGCAAGCCAGACACCAUAAGAGCUGUAAGGAUGAUAAAGAUGUCUUUCUACC
CUCGAGAGCUCUAAGUGUGGUCAGGAGAGAAACACAUGCAAAGAUUAUGCUGGCUGGAAG
GAAAUGCUGGAAUGAAGGUGGAAGCAUUCUAUUCAUCUAAAAGCACCGGUAAUUAACGAU
GCUAAUAAAUGUAUAAUCCCUUUGUAAACAUCUCUUCACAAGGUCAUAAAGAAGGAAGAC
UGAAUGAAGGACGCCAGGCUUUCAUUUUAGAAUUAAUUUUGUCAUUGAAUGCACUCACUG
CAUUAUUUGAUAGAGUACUUUUGCAUUUGAAGUAGUACAUUUGCCUUCUACCAUCUCAUU
AACAUUGCUUUCAACAGAAUCAGAAAUAACACAAACCACUGCAGGGUAAUUUUGAAUGCU
CAUUUGACUAUGCAGUGACAUCAAGUGGCGAGUUGAGGAAUGAUCAUAUGCUGUGUGAGA
ACUCCGUGUUAAAAAUUCCAUGGAGCCAGGCUAGACACCACAUUUGGCACUUUCAGCUGU
UUAGAUUUCAGAGAAUUAAAAGGAAAUGGUCACCGACCUGCCUUUGAUAGUACCCGUAUA
CCCCUUCCCACCAUAAGAAACCAGCGUUCCUUAGAGAAAUGGCUAAUUCUAGCUCUAGGG
CAAGAAAUGCUAAGAUGUGCUCAAAACAUCUCAUUAUACCAGCAAGCCAGGGAGCCAUCA
ACGACUACUGGAGCUGUGUCAAAAGGACCAACAAACCAACUUGAAGAGGCUCCCGCUGGC
CAACAGUGGGGCAAUUAGAGCAUCAAUAAGAAUAAUAUCUGUAACAAACUGAAACGUAUC
AAAUAUGUUUAAAUUCAUAUGUUUAUAAUUACACUCAAAAAACGAAGAUGCAGACCACUU
UACCUUGAGGAUGUUACAGAAUGAAAUUAUCAUUCUGAAAACUGGUAAAUAAAGAGAAGG
UAUCAAGUAAGCAUGUAUCUGGCUUUUUCUAUAUGAAAUAAAGGAAGCCAAAUAGUAAAC
GAGGGGAAGUUUCUCUUUACUGAAAUAUUGCAGGUAAUAAAAGAAGAAGGAGAAACAGAG
AACAUUAUCGUUUUAUGGCCUCUAAUGGAACAAGACAUCUAGGCAAUGAUGAACUAUGGU
CACCAACAUCACUCAAUCAGGGACAAUCAGACACUGUGGGCCCCUGAAGGAAGUAAACAC
CACCACGUAGGAAAUAUUCUCACCAAAACUUCAAAAACUUGAAUCUGAUCUAGUCUUUAG
UUUCAACUACUAAUUUACAGGCAAUACGGGGCUCAGAGGGAUGUGGUAAAAUGUCACGGA
AAUAUAAUCAGCAAAAUCCAGUACAGAUAAUAAGAAGACAAAUGAUCCACAGAUGGAAAG
GAAGAAUGUAAAGUUGUGUUGGUAAAGAAAUUCACAGAUUAAUAAAAGCUUAAAAAUGUA
CCA

Interaction probabilities

Prediction using RF classifier	0.7
Prediction using SVM classifier	0.98

What do these probabilities mean?

Interaction probabilities generated by RPISeq range from 0 to 1. In performance evaluation experiments, predictions with probabilities > 0.5 were considered "positive," i.e., indicating that the corresponding RNA and protein are likely to interact. Using this threshold, accuracies of the classifiers ranged from 87 - 90% in cross-validation evaluation experiments on benchmark datasets. When classifiers were tested on independent (blind) datasets of RPIs, accuracies of the classifiers ranged from 57 – 99%.
